# Transcriptome-Wide Annotation of m^5^C RNA Modifications Using Machine Learning

**DOI:** 10.3389/fpls.2018.00519

**Published:** 2018-04-18

**Authors:** Jie Song, Jingjing Zhai, Enze Bian, Yujia Song, Jiantao Yu, Chuang Ma

**Affiliations:** ^1^State Key Laboratory of Crop Stress Biology for Arid Areas, Center of Bioinformatics, College of Life Sciences, Northwest A&F University, Shaanxi, China; ^2^Key Laboratory of Biology and Genetics Improvement of Maize in Arid Area of Northwest Region, Ministry of Agriculture, Northwest A&F University, Shaanxi, China; ^3^College of Information Engineering, Northwest A&F University, Shaanxi, China

**Keywords:** AUC, Epitranscriptome, machine learning, RNA modification, RNA 5-methylcytosine

## Abstract

The emergence of epitranscriptome opened a new chapter in gene regulation. 5-methylcytosine (m^5^C), as an important post-transcriptional modification, has been identified to be involved in a variety of biological processes such as subcellular localization and translational fidelity. Though high-throughput experimental technologies have been developed and applied to profile m^5^C modifications under certain conditions, transcriptome-wide studies of m^5^C modifications are still hindered by the dynamic nature of m^5^C and the lack of computational prediction methods. In this study, we introduced PEA-m5C, a machine learning-based m^5^C predictor trained with features extracted from the flanking sequence of m^5^C modifications. PEA-m5C yielded an average AUC (area under the receiver operating characteristic) of 0.939 in 10-fold cross-validation experiments based on known *Arabidopsis* m^5^C modifications. A rigorous independent testing showed that PEA-m5C (Accuracy [Acc] = 0.835, Matthews correlation coefficient [MCC] = 0.688) is remarkably superior to the recently developed m^5^C predictor iRNAm5C-PseDNC (Acc = 0.665, MCC = 0.332). PEA-m5C has been applied to predict candidate m^5^C modifications in annotated *Arabidopsis* transcripts. Further analysis of these m^5^C candidates showed that 4nt downstream of the translational start site is the most frequently methylated position. PEA-m5C is freely available to academic users at: https://github.com/cma2015/PEA-m5C.

## Introduction

The epitranscriptome, also known as chemical modifications of RNA (CMRs), is a newly discovered layer of gene expression (Meyer and Jaffrey, 2014). With advances in mass spectrometry and high-throughput sequencing technologies, the field of epitranscriptome is rapidly expanding and attracting a comparable degree of research interests to DNA and histone modifications in the field of epigenetics (Helm and Motorin, 2017). Among more than 150 types of CMRs identified, most of them have been found in transfer RNAs (tRNAs) and ribosomal RNAs (rRNAs) (Hussain et al., 2013), but some can occur in mRNAs and noncoding RNAs (Machnicka et al., 2013; Pan, 2013; Carlile et al., 2014; Dominissini et al., 2016; David et al., 2017). A growing line of evidences indicated that CMRs located in both coding and noncoding regions can play essential roles in a variety of biological processes. For instance, N^6^-methyladenosine (m^6^A) sites in 5′-untranslated region (UTR) can promote cap-independent translation under heat stress (Meyer et al., 2015; Zhou et al., 2015); while m^6^A sites in coding regions can affect translation dynamics by inducing steric constraints and destabilizing pairing between codons and tRNA anticodons (Choi et al., 2016; Zhao et al., 2017). Thus, the transcriptome-wide annotation of RNA modifications is essential for fully understanding the biological functions of CMRs.

Compared with those well characterized modifications such as m^6^A and N^1^-methyladenosine (m^1^A), the transcriptome-wide annotation of 5-methylcytosine (m^5^C) modifications is more challenging. First, bisulfite sequencing technologies are difficult to implement for profiling m^5^C modifications because of the instability of mRNA molecules treated with bisulfite (Amort et al., 2013; Li et al., 2016). In addition, other existing high-throughput sequencing technologies, such as m^5^C-RIP (Edelheit et al., 2013), can localize m^5^C residues to transcript regions of 100–200 nucleotide (nt) long, but fail to accurately identify m^5^C modifications at single-nucleotide solution. Second, because of the dynamic nature of m^5^C (Wang and He, 2014), existing high-throughput sequencing technologies can only capture a snapshot of RNA modifications under certain experimental conditions, and cover just a small fraction of the whole transcriptome of a given sample (Zhou et al., 2016), resulting in the generation of significant numbers of false negatives (non-detected true m^5^C modifications). Third, the base preferences around the m^5^C sites are not strong enough, increasing the difficulties in computational predictions with traditional statistical approaches. Machine learning (ML) is a branch of artificial intelligence technology that has been widely used in engineering, computer science, informatics and biology (Ma et al., 2014a, 2017; Cui et al., 2015; Libbrecht and Noble, 2015; Zhai et al., 2016). The biggest advantage of ML systems is that they can automatically learn interesting patterns from existing datasets and bring about self-improvement of system performance for accurately predicting novel knowledge from a new data set (Ma et al., 2014a,b). Therefore, computational methods coupled with machine learning technologies may provide an option to accurately annotate RNA modifications like m^5^C in the transcriptome-wide manner.

Until now, iRNAm5C-PseDNC is the exclusive m^5^C predictor, which was built using random forest (RF) algorithm based on sequence-based features, and has been reported to have a good predictive performance for mammalian m^5^C prediction (Qiu et al., 2017). However, because of the lineage-specific sequence and structural properties differences between plant and mammalian species, tools developed for mammal species can't always retain their original performance when applied to other organisms (Leclercq et al., 2013; Zhai et al., 2017). This particular issue underscores the need for accurate transcriptome-wide m^5^C prediction tools in plants, which may lay a foundation for elucidating the mechanisms of formation and the cellular functions of m^5^C modifications.

In this study, we developed PEA-m5C, an accurate transcriptome-wide m^5^C predictor under a ML framework with an ensemble of 10 RF-based prediction models. PEA-m5C was trained with features extracted from the flanking sequence of m^5^C modifications, and showed promising performance when applied to predict m^5^C modifications in *Arabidopsis thaliana*. We further applied PEA-m5C to predict candidate m^5^C modifications in annotated *Arabidopsis* transcripts, and found that candidate m^5^C modifications are enriched in the coding region of mRNAs. In addition, 4-nt downstream of the translational start site is the most frequently methylated position. All candidate m^5^C modifications have been deposited in a public database named Ara-m5C for follow-up functional studies. In order to facilitate the application of PEA-m5C, we have implemented the proposed model into a cross-platform, user-friendly and interactive interface with R and JAVA programming languages.

## Materials and methods

### Dataset generation

In this study, we constructed four m^5^C datasets: DatasetCV (cross-validation dataset), DatasetHT (hold-out test dataset), DatasetIT1 (independent test dataset for samples from the *Arabidopsis* silique tissue) and DatasetIT2 (independent test dataset for samples from the *Arabidopsis* shoot tissue).

DatasetCV and DatasetHT were constructed based on m^5^C modifications in transcripts expressed in the *Arabidopsis* root tissue at single-nucleotide resolution using RNA bisulfite sequencing technology (David et al., 2017). During bisulfite conversion, unmethylated cytosines were converted into uracils, while methylated cytosines were not converted. Bisulfite-treated RNA samples were sequenced to generated 100-nt paired-end reads using the Illumina HiSeq 2500. Low-quality reads were processed using Trimmomatic (Bolger et al., 2014), and the left clean reads were globally mapped to *in silico* bisulfite-converted *Arabidopsis* reference genome sequences using the RNA mode of B-Solana (Kreck et al., 2012). For each cytosine site in the *Arabidopsis* reference genome, the methylation level was calculated using a proportion statistic: *P* = (C+Ψ)/(T+C), where C and T represent the number of cytosines and thymines in aligned reads at the cytosine site under analysis, respectively. Ψ specifies the added pseudo counts (1/8 counts). The false discovery rate (FDR) was calculated using the R package qvalue (Storey, 2002). Cytosines were regarded as positive samples (m^5^C modifications) if they satisfied the following criteria: methylation level ≥1% and FDR ≤ 0.3. After the removal of sequence redundancy, we finally obtained 1,296 m^5^C modifications in 885 transcripts (Table S1). In these 885 transcripts, cytosines were regarded as negative samples (non-m^5^C modifications) if they were not annotated as m^5^C modifications. In order to avoid over-fitting and GC bias in training process, we limited the number of negative samples to be 10 times of positive samples. Thus, for each positive sample, 10 samples were selected in the 200-nt region around the positive sample, among which GC content difference is not more than 5%. This allows a similar distribution of positive and GC-matched negative samples, which is markedly different from the background distribution of all cytosines in these 885 transcripts (Figure S1). Note that some of the negative samples may in fact be true m^5^C modifications not yet discovered. We randomly divided these 1,296 positive samples and 12,960 negative samples into two parts for constructing DatasetCV and DatasetHT, respectively. The DatasetCV comprises 1,196 positive samples and 11,960 negative samples, while the DatasetHT has a balanced number (100) of positive and negative samples (Table S1).

Using the same criteria mentioned above, another two datasets (DatasetIT1: 79 positive and negative samples; DatasetIT2: 73 positive and negative samples) were also constructed for *Arabidopsis* silique and shoot tissues, respectively (Table S1). Of note, positive and negative samples in DatasetIT1 and DatasetIT2 were not overlapped with those in DatasetCV and DatasetHT.

Each sample in these four datasets was represented by a sequence window of 43 nucleotides centered around the respective cytosine site. For samples near the borders of the available RNA sequence, the positions missing from the 43-nt window were filled with “N,” the symbol for unknown. The *Arabidopsis* reference genome sequences (TAIR10) and annotated transcripts used in this study were downloaded from the Araport 11 database (https://www.araport.org/data/araport11).

### Feature encoding

In order to be recognized by ML-based systems, each sample of *L*-nt window size, was represented as a numeric vector (length: 4^*^L + 106) using the binary, k-mer and PseDNC encoding schemes. The details of these three encoding schemes are described in the following.

#### Binary encoding

This encoding strategy generates a vector of 4^*^L features by characterizing “A,” “C,”, “G,” “U,” and “N” with (1, 0, 0, 0), (0, 1, 0, 0), (0, 0, 1, 0), (0, 0, 0, 1), and (0, 0, 0, 0) for each sample, respectively.

#### K-mer encoding

In this scheme, the composition of short sequence with different lengths was considered to explore its potential effect on the identification of m^5^C. In order to avoid the curse of dimensionality, we set *k* = 1, 2, and 3 to generate 84 features for calculating the frequency of mononucleotide occurrence (*k* = 1; four features), dinucleotide occurrence (*k* = 2; 16 features) and trinucleotide occurrence (*k* = 3; 64 features).

#### PseDNC encoding

The pseudo dinucleotide composition (PseDNC) is a widely used encoding strategy that considers sequential information as well as physicochemical properties of dinucleotides in the RNA sequence (Chen et al., 2015, 2017). For each sample, it generates 16+λ numeric features, the first 16 of which are features extracted from adjacent dinucleotide pairs, and the other λ are features extracted from distant dinucleotide pairs (λ denotes the maximal distance between two dinucleotides). The detailed definition of PseDNC is presented in Supplementary Data 1.

### Development of ML-based m^5^C predictor

**Figure 1:**
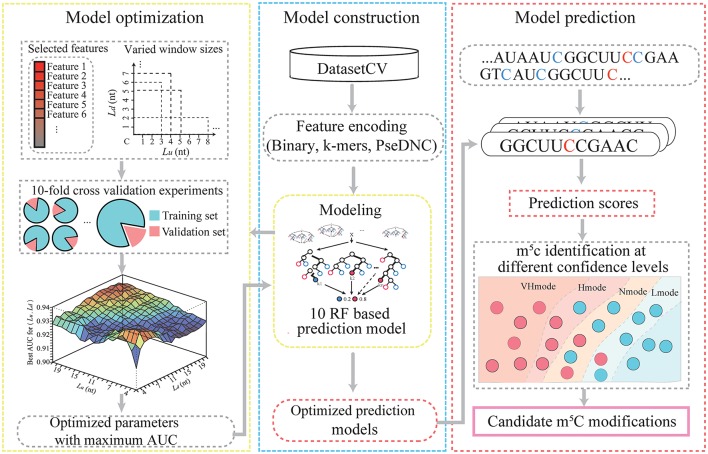
The computational framework of PEA-m5C.

Figure 1 illustrates the workflow of PEA-m5C, which consists of three phases, namely, (A) model construction, (B) model optimization, and (C) model prediction. Model construction and optimization were performed on the DatasetCV.

#### Model construction

To construct an m^5^C prediction model, PEA-m5C required an input of a set of positive and negative samples. These samples were transformed into a feature matrix using three different encoding schemes (binary, k-mer, and PseDNC). The feature matrix was input into the RF algorithm to construct an m^5^C prediction model, which consisted of 100 classification trees. Each of the classification trees was built using a set of bootstrapped samples and features. The output of the RF-based m^5^C prediction model was determined by a majority vote of the classification trees. The RF algorithm was implemented using the R package “Rweka” (Hornik et al., 2009), which provides an R environment to invoke the ML package “weka” (v3.9.1; https://www.cs.waikato.ac.nz/ml/weka).

#### Model optimization

Ten-fold cross-validation experiments were performed to optimize m^5^C prediction models in PEA-m5C by iteratively varying window size and feature number. Cross-validation is a standard method for estimating the generalization accuracy of ML systems. In a ten-fold cross-validation, the DatasetCV was randomly divided into 10 equal subsets and each subset was iteratively selected as a testing set for evaluating the model trained with other nine subsets. In each fold of cross-validation, considering the high unbalance between positive and negative samples (1:10), the negative samples were randomly divided into 10 parts, each of which coupled with the set of positive samples were used for training an RF-based m^5^C prediction model. Therefore, ten RF-based m^5^C prediction models were constructed in the training process. In the testing process, each sample was scored using these ten RF-based m^5^C prediction models. The corresponding ten prediction scores were averaged as the final prediction score of the sample under analysis. Once the testing process was completed, the prediction accuracy of PEA-m5C (an ensemble of ten RF-based m^5^C prediction models) was evaluated using the receiver operating characteristic (ROC) analysis, which plots a curve of false positive rate (FPR) varying at different true positive rate (TPR). The value under the ROC curve (AUC) was used to quantitatively score the prediction performance of PEA-m5C. AUC is ranged from 0 to 1, the higher the better prediction performance. After 10 subsets have been successively used as the testing set, the corresponding 10 AUC values were averaged as the overall prediction performance of PEA-m5C.

**Figure 2:**
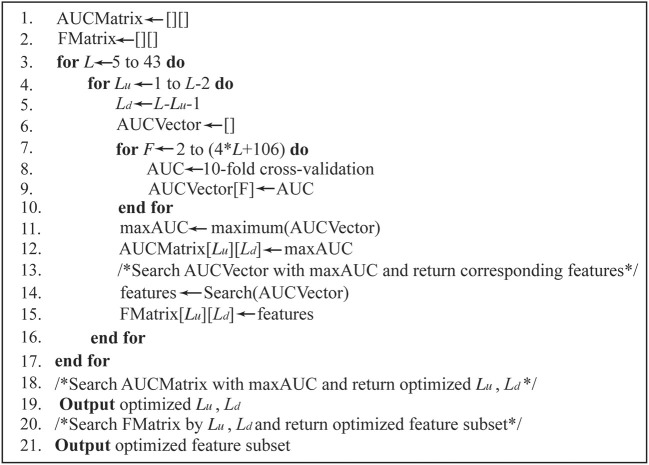
The pseudo-code for model optimization.

The PEA-m5C was optimized to maximize the AUC by iteratively varying window size *L* from 5- to 43-nt and feature number from 2 to 4^*^L+106. The feature subset was selected according to the feature importance estimated using the information gain approach implemented in R package “FSelector” (Cheng et al., 2012). The detailed process of model optimization is given in Figure 2. We initialize AUC matrix (“AUCMatrix”) and feature matrix (“FMatrix”) as two empty sets (**Lines 1-2**). Then for a given window size *L* (5-nt ≤ *L* ≤ 43-nt) (**Line 3**), we varied the upstream sequence length (*L*_*u*_) from 1-nt to (*L*-2)-nt and the number of feature subset from 2 to 4^*^L+106 (**Lines 4-7**). Subsequently, for each feature subset, we performed a 10-fold cross-validation experiment and stored the corresponding AUC value into a vector (“AUCVector”) (**Lines 8-9**). After all possible feature subsets have been examined using 10-fold cross-validation experiments, the maximum AUC in “AUCVector” will be stored in the “AUCMatrix” (**Lines 11-12**), and the corresponding feature subset with maximum AUC will be stored in “FMatrix” (**Lines 13-15**). Finally, after all possible window sizes have been performed, the optimized *L*_*u*_ and *L*_*d*_ can be obtained by searching the maximum value in “AUCMatrix” **(Lines 18-19**), and the optimized feature subset can be obtained by searching “FMatrix” with *L*_*u*_ and *L*_*d*_ (**Lines 20-21**).

#### Model prediction

PEA-m5C predicted all candidate m^5^C modifications in given RNA sequences in FASTA format. For each cytosine site, PEA-m5C firstly extracted the flanking sequence with the optimized window size. Then, three feature encoding schemes were performed to transform the flanking sequence to a numeric vector. Subsequently, the optimized feature subset was input into the ten RF-based m5C prediction models. Finally, PEA-m5C generated a prediction score to reflect the possibility of this cytosine to be a real m^5^C modification. Of note, four thresholds have been also included in the PEA-m5C, which were automatically determined in the 10-fold cross-validation at the specificity level of 99, 95, 90, and 85%, respectively. These four thresholds corresponded to four different confidence modes of PEA-m5C: VHmode (very high confidence mode), HMode (high confidence mode), NMode (normal confidence mode) and LMode (low confidence mode), respectively. Cytosine sites with a prediction score higher than the threshold were predicted as positive samples; otherwise, they were predicted as negative samples.

### Model comparisons

The iRNAm5C-PseDNC is only available m^5^C predictor that aims to accurately predict m^5^C modifications in mammalian genomes. It was constructed using the RF algorithm with only PseDNC features, and was trained with mammalian m^5^C modifications (window size: 41-nt) (Sun et al., 2016). In order to fairly compare prediction performance between iRNAm5C-PseDNC and our proposed model PEA-m5C, we also re-trained iRNAm5C-PseDNC with positive and negative samples of 41-nt in the DatasetCV, and this re-trained predicted model was named as iRNAm5C-PseDNC^*^. Prediction performance of iRNAm5C-PseDNC, iRNAm5C-PseDNC^*^ and PEA-m5C was estimated on DatasetHT, DatasetIT1 and DatasetIT2 using six widely used measures: sensitivity (Sn, also known as recall), specificity (Sp), precision (Pr), accuracy (Acc), F_1_-score (F_1_), and Matthews correlation coefficient (MCC). These measures were defined as follows:

$$\begin{array}{l} \text{Sn}=\;\frac{\text{TP}}{\text{TP}+\text{FN}},\\ \text{Sp}=\;\frac{\text{TN}}{\text{TN}+\text{FP}},\\ \text{Pr}=\;\frac{\text{TP}}{\text{TP}+\text{FP}},\\ \text{Acc}=\;\frac{\text{TP}+\text{TN}}{\text{TP}+\text{TN}+\text{FP}+\text{FN}},\\ {\text{F}}_{1}=\frac{2*\mathrm{Pr}\times \text{Sn}}{\text{Pr}+\text{Sn}}=\frac{2*\text{TP}}{2*\text{TP}+\text{FP}+\text{FN}},\\ \text{MCC}=\;\frac{\text{TP}*\text{TN}-\text{FP}*\text{FN}}{\sqrt{\left(\text{TP}+\text{FP}\right)*\left(\text{TP}+\text{FN}\right)*\left(\text{TN}+\text{FP}\right)*\left(\text{TN}+\text{FN}\right)}}, \end{array}$$

where TP, TN, FP, and FN represent the number of true positives, true negatives, false positives and false negatives, respectively. F_1_ is the harmonic mean of Pr and Sn. Compared with Sn, Sp, Pr, and F_1_, Acc and MCC are two more information measures which combine all of the predictions (TP, TN, FP, and FN) into a single score. Acc, which ranges from 0 to 1, measures the proportion of correct predictions. MCC, also known as the phi coefficient, measures the correlation between the observations and predictions. It is generally regarded as a balanced measure, which can be used even if the two classes are of very different size. The value of MCC ranges from −1 to 1, where 1 represents a perfect prediction, 0 indicates no better than random prediction and −1 means total disagreement between observations and predictions.

### Transcriptome-wide m^5^C annotation and analysis

Candidate m^5^C sites in the annotated *Arabidopsis* transcripts were predicted using the PEA-m5C. The spatial distribution of candidate m^5^C modifications was statistically analyzed in three aspects: (i) feature enrichment (e.g., 5′-UTR, coding region [CDS] and 3′-UTR) analysis of candidate m^5^C modifications in coding RNAs; (ii) the most frequently methylated position relative to the translational start site; (iii) functional enrichment analysis of genes containing candidate m^5^C modifications.

The base preference around candidate m^5^C modification sites was also explored, including: (i) the proportion of m^5^C modifications in different sequence contexts: CG, CHG and CHH (H: A, T or C); (ii) sequence motifs of candidate m^5^C modifications.

## Results

### Characterization of m^5^C modifications using sequence-based features

**Figure 3:**
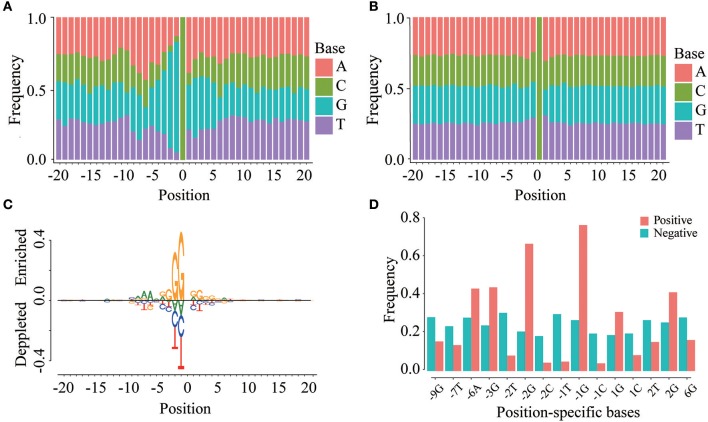
Position-specific base usage in positive and negative samples. The region around (−20, 20) around the cytosine sites is used to perform position-specific base usage analysis, where the cytosines are defined as 0. **(A)** Frequencies of 41 × 4 position-specific bases in positive samples. **(B)** Frequencies of 41 × 4 position-specific bases in negative samples. **(C)** Two sample logo of the differences between m^5^C and non-m^5^C modifications. It shows nucleotides which are enriched or depleted in the surrounding regions of m^5^C modifications. **(D)** With a significance level of 1.0E-10, the usage of 15 position-specific bases is significantly different between m^5^C and non-m^5^C modifications.

To investigate whether m^5^C modifications can be identified using sequence-based features, we first examined the positional frequencies of four bases in positive and negative samples in the DatasetCV (Figures 3A,B). We observed that the positional base frequency appears to be stable in negative samples. In contrast, the positional base frequency was biased to guanine (G) in the region near m^5^C sites in positive samples. We then detected position-specific base usages by using rank sum test. Setting significant level (*p*-value) to be 1.0E-10, we found that 15 position-specific base usages are significantly different between positive and non-m^5^C modifications. They are −9G,−7T,−6A,−3G,−2T,−2G,−2C,−1T,−1C, 1G, 1C, 2T, 2G, 6G. The difference can be visualized by comparing the frequencies of these position-specific bases in m^5^C and non- m^5^C modifications (Figure 3C). Furthermore, through two sample logo analysis using R package “DiffLogo” (Nettling et al., 2015), we discovered the similar trend of some specific nucleotide usage preferences around m^5^C modifications (Figure 3D). These results indicate that base frequency differences exist between m^5^C and non- m^5^C modifications.

**Figure 4:**
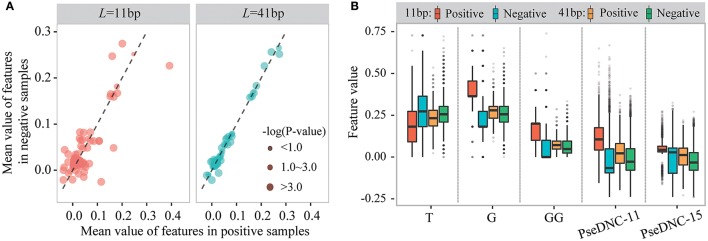
The different distribution of k-mer and PseDNC features between positive and negative samples. **(A)** The differences of k-mer and PseDNC features between positive and negative samples based on the mean value difference and two-sample *t*-test. **(B)** The feature distribution is different between the positive and negative samples and affected by the window size.

We then examined sequence-based features generated from k-mer and PseDNC encoding schemes. Figure 4A displays the mean values of these features for positive and negative samples. When the window size is 11-nt (*L*_*u*_ = *L*_*d*_ = 5), we detected 70 k-mer-based features and 19 PseDNC-based features significantly different between positive and negative samples (two-sample *t*-test; *p* ≤ 1.0E-4). The top-five ranked features are the frequency of T, G, GG and PseDNC-11, PseDNC-15 (Figure 4B). When the window size was extended from 11-nt to 41-nt (*L*_*u*_ = *L*_*d*_ = 20), we also detected 32 k-mer-based features and 12 PseDNC-based features at the significance level of 1.0E-4. The top-five ranked features are the frequency of G, GG and GGC, PseDNC-11 and PseDNC-15 (Figure 4B).

Taken together, these results indicate that the three encoding schemes, binary, k-mer and PseDNC, can generate discriminative features for m^5^C prediction. However, the importance of different features is affected by the window size used.

### A machine learning-based m^5^C predictor with optimized window size and features

**Figure 5:**
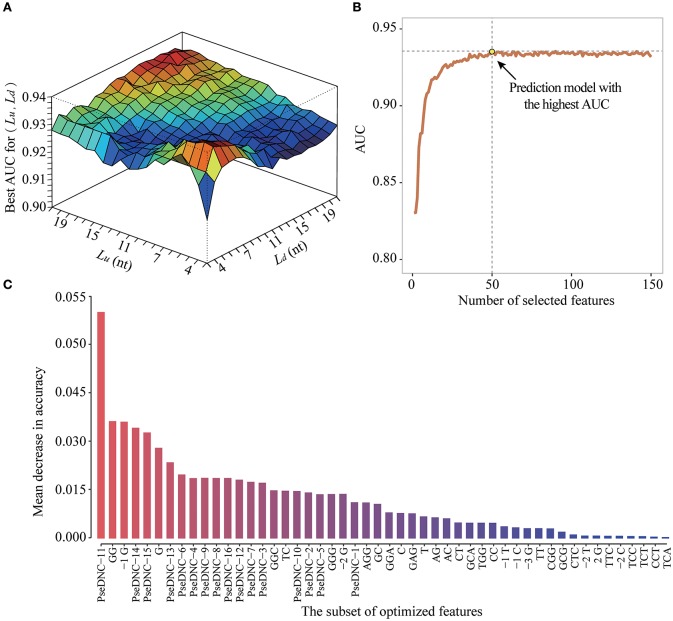
The performance improvement of PEA-m5C using a hybrid optimization strategy. **(A)** The prediction performance of PEA-m5C in terms of AUC is optimized by varying window size and feature number. **(B)** The prediction performance of PEA-m5C in terms of AUC is optimized with different number of features for a given window size. **(C)** The top 50-ranked features used in the optimized prediction models of PEA-m5C.

To obtain the optimized window size and feature subset, we iteratively performed ten-fold cross-validation experiments on the DatasetCV by varying window size *L* from 5-nt to 43-nt and the feature number *F* from 2 to 106+4^*^L (Figure 5A). For a given window size of *L* (e.g., upstream region: *L*_*u*_ = 10 and downstream region: *L*_*d*_ = 5) and feature number of *F* (e.g., *F* = 50), we performed a 10-fold cross-validation experiment to calculate an AUC value for evaluating the prediction performance of PEA-m5C. Then, at the given window size *L*, the best AUC value achieved by PEA-m5C can be found according to the curve depicted in Figure 5B, where *x* axis represents the number of selected features and *y* axis represents the AUC yielded by PEA-m5C. After examining all possible combinations of window sizes and feature numbers, we observed that PEA-m5C achieved the highest AUC value of 0.939 (Figure 5A), when the window size was set as 11-nt (*L*_*u*_ = *L*_*d*_ = 5) and 50 top ranked features were used (Figure 5C,Table S2).

### Prediction evaluation and comparison using hold-out and independent testing sets

**Figure 6:**
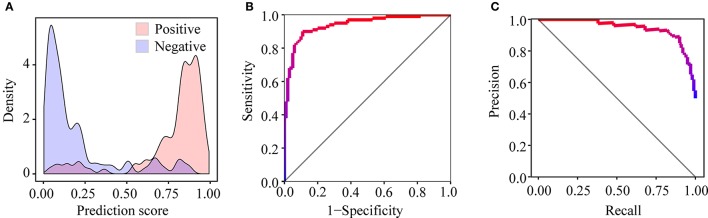
Performance evaluation of PEA-m5C on the DatasetHT. **(A)** The different distribution of prediction scores for positive and negative samples. **(B)** The ROC curve illustrating the high performance of PEA-m5C. **(C)** The precision-recall curve illustrating the high performance of PEA-m5C.

After training PEA-m5C using the DatasetCV with the optimized window size and feature subset, we next evaluated the performance of PEA-m5C on a hold-out test set (DatasetHT). As shown in Figure 6A, the prediction score of positive samples (mean ± standard deviation [sd]: 0.775 ± 0.223) was significantly higher than that of negative samples (mean ± sd: 0.194± 0.225). This result indicates that PEA-m5C could provide a competitive performance in discriminating positive and negative samples. Indeed, PEA-m5C gave an area under ROC (AUC) and an area under the precision-recall curve (auPRC) of 0.939 and 0.945, respectively (Figures 6B,C). To assess the performance more comprehensively, six measures (Sn, Sp, Pr, Acc, MCC, and F_1_) were examined at four thresholds, corresponding to the specificity level of 99% (very high confidence mode; VHmode), 95% (high confidence mode; HMode), 90% (normal confidence mode; NMode) and 85% (Low confidence mode; LMode) in the 10-fold cross-validation experiment, respectively (Table 1). In line with the intuitive observations of ROC curve (Figure 6B) and precision-recall curve (Figure 6C), PEA-m5C performed markedly better than random selection (AUC = 0.5, auPRC = 0.5, and MCC = 0) in predicting m^5^C modifications at four different specificity levels (Table 1).

**Table 1:** Prediction performance of m^5^C predictors on DatasetHT.

**m^5^C predictor**	**Mode**	**Threshold**	**Sn**	**Sp**	**Pr**	**Acc**	**MCC**	**F_1_**
PEA-m5C	VHmode	0.891	0.330	1.000	1.000	0.665	0.445	0.496
	HMode	0.765	0.720	0.950	0.935	0.835	0.688	0.814
	NMode	0.622	0.860	0.900	0.896	0.880	0.761	0.878
	LMode	0.484	0.900	0.860	0.865	0.880	0.761	0.882
iRNAm5C-PseDNC (web server)	–	–	0.010	0.980	0.333	0.495	−0.041	0.019
iRNAm5C-PseDNC (Method)^*^	–	0.500	0.610	0.720	0.685	0.665	0.332	0.646

* *An m5C prediction model generated by re-training iRNAm5C-PseDNC using positive and negative samples from the DatasetCV*.

Currently, iRNAm5C-PseDNC is the only software available for m^5^C prediction; however, it was built based on mammalian m^5^C modifications. This provides us an opportunity to evaluate whether iRNAm5C-PseDNC could retain prediction accuracy on *Arabidopsis* m^5^C modifications. We observed that iRNAm5C-PseDNC yielded a high specificity of 0.980, but an extremely low sensitivity of 0.010. The main reason is that there are significant differences between mammalian and *Arabidopsis* m^5^C modifications (Figure S2). To examine the effectiveness of ML algorithms in iRNAm5C-PseDNC, we generated a new prediction model (named as iRNAm5C-PseDNC^*^) by re-training iRNAm5C-PseDNC using positive and negative samples from the DatasetCV and evaluated its performance using the DatasetHT. Compared with iRNAm5C-PseDNC, iRNAm5C-PseDNC^*^ yielded higher prediction accuracy at the level of Sn, Sp, Pr, Acc, MCC, and F_1_. However, PEA-m5C still achieved higher prediction accuracy than iRNAm5C-PseDNC and iRNAm5C-PseDNC^*^ (Table 1). The prediction performance of PEA-m5C was also better than iRNAm5C-PseDNC and iRNAm5C-PseDNC^*^ on DatasetIT1 and Dataset2, which consist of samples from *Arabidopsis* silique and shoot tissues, respectively (Table S3).

Taken together, these results indicate that the construction of *Arabidopsis thaliana*-specific predictor is necessary and crucial. In addition, PEA-m5C is a useful tool for the prediction of m^5^C sites in *Arabidopsis* transcripts.

### Transcriptome-wide annotation and analysis of candidate m^5^C modifications

The encouraging performance of PEA-m5C in the cross-validation and validation testing experiments provide us an opportunity to accurately predict m^5^C sites in the annotated *Arabidopsis* transcripts. At the threshold of 0.891 (VHMode), PEA-m5C predicted 303,421 candidate m^5^C modifications (Table 2), covering 4.56% cytosines (303,421/6,650,570) in all annotated transcripts in Araport 11 database (https://www.araport.org/data/araport11). During the writing of our manuscript, Cui and colleagues identified 4,439 m^5^C peaks in 3,534 expressed genes (Table S4) in young seedlings of *Arabidopsis* (Cui et al., 2017), by applying m^5^C RNA immunoprecipitation followed by a deep-sequencing approach. We validated the m^5^C predictions using these 4,439 m^5^C peaks. Among the 3,534 expressed genes, PEA-m5C identified 5,463 candidate m^5^C modifications, covering 2,724 of 4,439 reported peak regions. We note that the proportion of covered m^5^C peaks increased from 61.4% (2,724/4,439) to 89.4% (3,968/4,439), when the HMode was used.

**Table 2:** Candidate m^5^C modifications in different types of RNAs. Num: number, Prop: proportion, trans: transcripts.

**RNA**	**Num of trans**	**Num of cytosines**	**Num (Prop) of cytosines are methylated**	**Num (Prop) of trans containing m^5^C**
Long noncoding RNA	2,455	161,608	1,480 (0.92%)	967(39.39%)
miRNA	387	1,082	15 (1.29%)	12(3.10%)
Primary miRNA transcript	325	9,717	100 (1.03%)	74(22.77%)
mRNA	48,353	16,727,847	225,348 (1.35%)	44,350(91.72%)
rRNA	15	4,041	147 (3.64%)	12(80.00%)
snoRNA	287	5,049	70 (1.39%)	55(19.16%)
snRNA	82	2,906	62 (2.13%)	35(42.68%)
tRNA	689	10,227	272 (2.66%)	232(33.67%)

**Figure 7:**
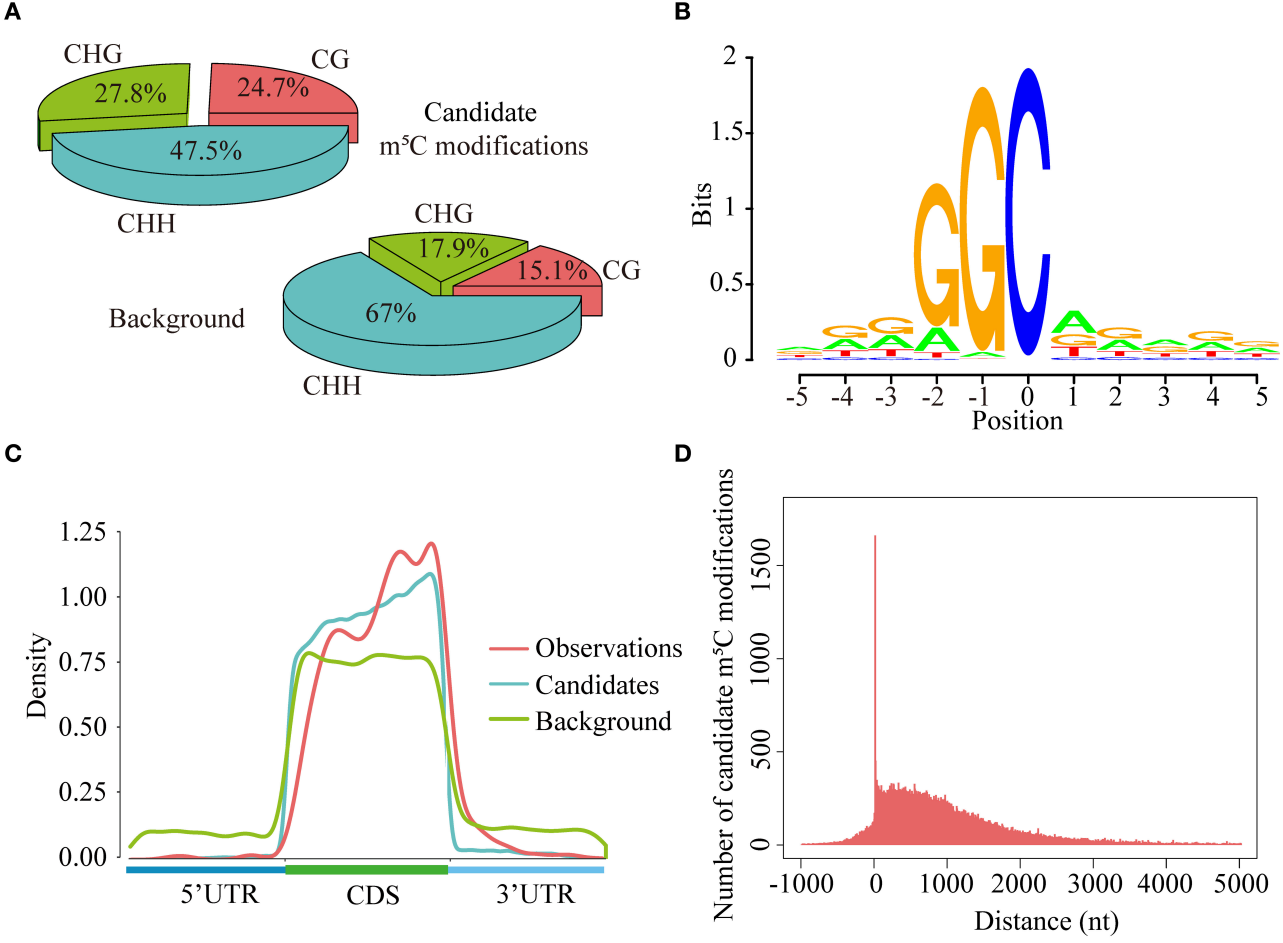
Transcriptome-wide annotation of m^5^C modifications using PEA-m5C. **(A)** The proportions of candidate m^5^C modifications and all cytosine sites in three different sequence contexts: CG, CHG and CHH (where H = A, C, or U). **(B)** The sequence logo of candidate m^5^C modifications. The position of candidate m^5^C modifications is defined as 0. **(C)** The distribution of observed m^5^C modifications (positive samples in the DatasetCV), candidate m^5^C modifications, and all cytosine sites (background) along the 5′-UTR, CDS and 3′-UTR, normalized for transcript length. **(D)** The distribution of candidate m^5^C modifications relative to translational start sites. The position of translational start sites is defined as 0.

As is known to us all, cytosines in DNA sequences can be methylated in three sequence context, namely CG, CHG, and CHH (H = A, C, or T) (Smith and Meissner, 2013). In this study, we explored the levels of cytosine methylation in RNA sequences. We observed that 24.7, 27.8, and 47.5% of the candidate m^5^C modifications are methylated in the CG, CHG, and CHH sequence context, respectively. These proportions are markedly different from those of cytosines in background sequences (CG: 15.1%, CHG: 17.9%, CHH: 67.0%) (Figure 7A). Statistical analysis of base preference showed that there are very strong “G” signal around candidate m^5^C modifications (Figure 7B). These results indicate that candidate m^5^C modifications predicted by PEA-m5C may have potential biological functions.

Toward a better understanding of these candidate m^5^C modifications, we further analyzed the enrichment of m^5^C within three different regions of mRNAs: 5′-UTR, CDS and 3′-UTR. It can be seen from Figure 7C that the majority of m^5^C modifications are located in CDS regions. Recent studies have indicated that the m^5^C modification prefers to occur at the downstream of translational start sites in mammal mRNAs (Amort et al., 2017; Yang et al., 2017). We calculated the distance between candidate m^5^C modifications and translational start sites, and found that the most frequently m^5^C modification position is the 4nt downstream of the translational start site (AUG^*^C**;** methylated cytosines are in bold and underlined) (Figure 7D). In order to further investigate the potential function of those 1,063 genes with m^5^C modifications located at 4-nt downstream of the translational start site, we performed a GO (gene ontology) enrichment analysis using agriGO 2.0 (Tian et al., 2017) and found that in the BP (Biological Progress) sub-category, 166 genes (Table 3) are enriched in the term “response to stimulus” with FDR of 2.40E-4; For the MF (Molecular Function) sub-category, 350 genes are significantly enriched in “catalytic activity” with FDR of 9.80E-07 (Table 3). We also performed pathway enrichment analysis on these 1063 genes using the hypergeometric distribution test. Pathway information was obtained from KEGG (http://www.genome.jp/kegg) and AraCyc (http://www.plantcyc.org) databases. At the level of *p* ≤ 1.0E-2, we identified four significantly enriched pathways, including L-lysine biosysthesis VI pathway, glutathione metabolism, N-Glycan biosynthesis, and phosphatidylinositol signaling system (Table S5).

**Table 3:** Top five significant GO terms in the sub-category of biological progress (BP), molecular function (MF), and cellular component (CC).

**GO Term**	**Enriched gene number**	**FDR**	**Category**
Lipid localization	10	2.60E-05	BP
Response to stimulus	166	0.00024	BP
Macromolecule localization	34	0.00032	BP
Localization	91	0.00032	BP
Toxin metabolic process	11	0.00032	BP
Transporter activity	91	3.70E-09	MF
Transmembrane transporter activity	69	9.80E-07	MF
Catalytic activity	350	9.80E-07	MF
Substrate-specific transporter activity	63	6.90E-06	MF
Substrate-specific transmembrane transporter activity	56	1.10E-05	MF
Cytoplasm	299	2.60E-14	CC
Cell part	548	1.00E-13	CC
Cell	548	1.00E-13	CC
Cytoplasmic part	276	1.40E-13	CC
Membrane	200	1.40E-13	CC

### Implementation of PEA-m5C

**Figure 8:**
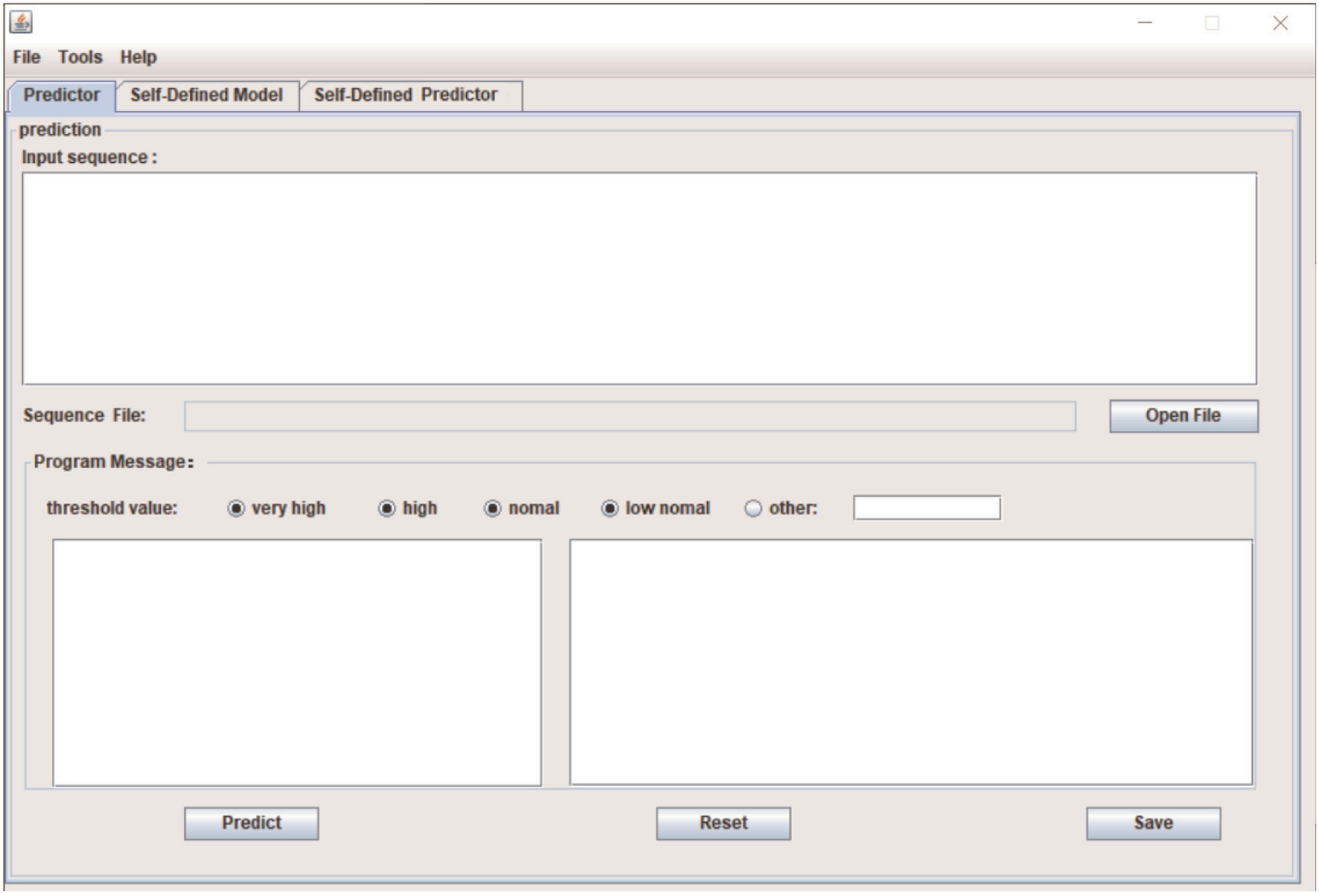
The Java interface implementation of PEA-m5C.

To facilitate the practicability, we implemented PEA-m5C into an R package named “PEA-m5C”. We also provided a cross-platform, user-friendly and interactive interface for PEA-m5C with JAVA programming language (Figure 8). This allows the user to easily implement PEA-m5C without the requirement of any programming skills or knowledge. To expand the application of PEA-m5C to other species, users can also retrain prediction models through the pre-specified dataset using the “Self-Defined Mode” option in PEA-m5C, with the input of positive and negative samples in FASTA format. PEA-m5C is freely available to academic users at: https://github.com/cma2015/PEA-m5C.

## Discussion

In this study, we developed PEA-m5C, a computationally framework for accurate identification of m^5^C modifications in *Arabidopsis*. PEA-m5C predictor was constructed using RF algorithm with optimized window size and sequence-based features, achieving a considerable promising performance no matter from 10-fold cross-validation experiment or hold-out test experiment. The PEA-m5C is superior to the newly developed and only available m^5^C predictor iRNAm5C-PseDNC in several aspects.

First, besides the PseDNC encoding scheme used in iRNAm5C-PseDNC, PEA-m5C additionally integrates another two encoding schemes (binary and k-mer) to make more use of sequence-based features. Both 10-fold cross-validation and independent testing experiments have demonstrated that higher prediction accuracy can be achieved by PEA-m5C when more feature encoding schemes were used (Figure S3; Table S6). For instance, in the 10-fold cross-validation, PEA-m5C yielded an AUC of 0.904, 0.914 and 0.939 when PseDNC, PseDNC + k-mer, PseDNC + k-mer + binary encoding schemes were used, respectively.

Second, PEA-m5C uses a hybrid optimization strategy to produce better prediction accuracy (Table S6), while iRNAm5C-PseDNC didn't perform the model optimization process. This is understandable as the model optimization is a rather timing-consuming process (Figure 2). However, the results shown in Figure 5 illustrated the importance of model optimization in developing accurate m^5^C predictors. We also would like to note that the process of model optimization requires to be finely tuned, such as the choice of appropriate feature selection approaches. To select informative features for m^5^C prediction, we preferred to use the information gain approach rather than statistical analysis approaches (e.g., chi-square test for binary features, student's *t*-test for k-mer- and PseDNC-based features). While testing on the DatasetHT, PEA-m5C using the information gain approach yielded a slightly higher maximum MCC (0.790) than that using the chi-square test and the student's *t*-test (0.770).

Finally, PEA-m5C has been implemented into a user-friendly interface with JAVA programming language and an R package to maximize its practicality. It also includes a self-training module that provides an option to automatically build m^5^C predictors for specific species, tissues, or conditions. This is very important as m^5^C modifications exhibit different sequence patterns in different issues (Figure S4).

In the future, we will endeavor to incorporate more features (e.g., structure-based features) to further improve the performance of PEA-m5C. If possible, specie-specific or tissue-specific predictors will be developed to facilitate the functional investigation of m^5^C modifications in plants.

## Author contributions

CM: Designed the experiments; JS, JZ, and EB: Performed the experiments; JS, JZ, EB, CM, JY, and YS: Analyzed the data; CM, JZ, and JS: Wrote the paper. All authors read and approved the final manuscript.

### Conflict of interest statement

The authors declare that the research was conducted in the absence of any commercial or financial relationships that could be construed as a potential conflict of interest.

## Supplementary material

Supplementary Data 1 The description of PseDNC encoding.

Figure S1 Location distribution of positive, negative and background samples along the 5′-UTR, CDS and 3′-UTR, normalized for transcript length (relative distance).

Figure S2 Two sample logos of *Arabidopsis* and mammalian m^5^C modifications. It shows nucleotides which are enriched or depleted in the surrounding region of m^5^C modifications.

Figure S3 The ROC curve of 10-fold cross-validation illustrating the performance of PEA-m5C with different feature encoding schemes.

Figure S4 Different sequence patterns of m5C modifications in DatasetHT, DatasetIT1, and DatasetIT2. **(A)** Frequencies of 41 ^*^ 4 position-specific bases in DatasetHT (Root tissue) and DatasetIT1 (Silique tissue). **(B)** Two sample logos DatasetHT (Root tissue) and DatasetIT1 (Silique tissue) m^5^C modifications. **(C)** Frequencies of 41 ^*^ 4 position-specific bases in DatasetHT (Root tissue) and DatasetIT2 (Shoot tissue). **(D)** Two sample logos DatasetHT (Root tissue) and DatasetIT2 (Shoot tissue) m^5^C modifications.

Table S1 Four benchmark datasets constructed for the prediction of m^5^C modifications in this study.

Table S2 The feature importance measured using the information gain approach at the window size of 11-nt (*L*_*u*_ = *L*_*d*_ = 5).

Table S3 Prediction performance of m^5^C predictors on DatasetIT1 and DatasetIT2.

Table S4 Peak regions used for validating transcriptome-wide candidate m^5^C modifications predicted by PEA-m^5^C.

Table S5 Enriched pathways of genes containing m^5^C modifications at 4-nt downstream of the translational start site.

Table S6 The performance of m^5^C predictors on DatasetHT using different encoding schemes.
