# Corrigendum: Transcriptome-Wide Annotation of m^5^C RNA Modifications Using Machine Learning

**DOI:** 10.3389/fpls.2018.01762

**Published:** 2018-11-30

**Authors:** Jie Song, Jingjing Zhai, Enze Bian, Yujia Song, Jiantao Yu, Chuang Ma

**Affiliations:** ^1^State Key Laboratory of Crop Stress Biology for Arid Areas, Center of Bioinformatics, College of Life Sciences, Northwest A&F University, Shaanxi, China; ^2^Key Laboratory of Biology and Genetics Improvement of Maize in Arid Area of Northwest Region, Ministry of Agriculture, Northwest A&F University, Shaanxi, China; ^3^College of Information Engineering, Northwest A&F University, Shaanxi, China

**Keywords:** AUC, Epitranscriptome, machine learning, RNA modification, RNA 5-methylcytosine

In the original article, there was an error, the word “reversible” is misleading. A correction has been made to the Abstract and the Introduction, paragraph 2.

Though high-throughput experimental technologies have been developed and applied to profile m^5^C modifications under certain conditions, transcriptome-wide studies of m^5^C modifications are still hindered by the dynamic nature of m^5^C and the lack of computational prediction methods.

Second, because of the dynamic nature of m^5^C (Wang and He, 2014), existing high-throughput sequencing technologies can only capture a snapshot of RNA modifications under certain experimental conditions, and cover just a small fraction of the whole transcriptome of a given sample (Zhou et al., 2016), resulting in the generation of significant numbers of false negatives (non-detected true m^5^C modifications).

The authors apologize for the mistake. This error does not change the scientific conclusions of the article in any way. The original article has been updated.

## Conflict of interest statement

The authors declare that the research was conducted in the absence of any commercial or financial relationships that could be construed as a potential conflict of interest.
