# Supplementary material for: Transcriptome-Wide Annotation of m5C RNA Modifications Using Machine Learning
Source: Front Plant Sci. 2018 Apr 18;9:519. doi: 10.3389/fpls.2018.00519 (PMC5915569; doi:10.3389/fpls.2018.00519)
Supplement: Supplementary Data 1 — The description of PseDNC encoding. [file Presentation1.PDF]

Supplementary Material

Transcriptome-wide Annotation of m^{5}C RNA Modifications Using Machine Learning

Jie Song[1,2,#] , Jingjing Zhai[1,#], Enze Bian[3,#], Yujia Song[3], Jiantao Yu[3],Chuang Ma[1,2 *]

*** Correspondence:** Chuang Ma: chuangma2006@gmail.com

1 Supplementary Data

The Description of PseDNC (Pseudo Dinucleotide Composition)

The pseudo dinucleotide composition (PseDNC) is a widely used encoding strategy that integrates the local and global sequence pattern information along the RNA sequence (Chen et al., 2015). For a given sample, it can be transformed into a (16+λ)-dimensional vector shown as follows:

$$R = [d_1, d_2 \ldots d_{16}, d_{16+1} \ldots d_{16+\lambda}]^{\mathrm{T}},$$

The first 16 components are features extracted from adjacent dinucleotide pairs, and the other λ components are features extracted from distant dinucleotide pairs (λ denotes the maximal distance between two dinucleotides).

For the *m*th component in *R*, it is defined using the following formula:

$$d_{\mathrm{m}} = \begin{cases} \frac{\mathrm{f_m}}{\sum_{i=1}^{16} \mathrm{f_i} + w\sum_{j=1}^{\lambda} \theta_j}, & 1 \le \mathrm{m} \le 16 \\ \frac{w\theta_{m-16}}{\sum_{i=1}^{16} \mathrm{f_i} + w\sum_{j=1}^{\lambda} \theta_j}, & 16+1 \le \mathrm{m} \le 16+\lambda \end{cases},$$

where *w* is the weight factor which defines the proportion of global sequence information relative to local sequence pattern information, and $\mathrm{f_m}$ represents the normalized frequency of different dinucleotides. θ_j is the correlation factor between dinucleotides with a certain distance *j* of interval along a RNA sequence, which can be calculated as:

$$\theta_j = \frac{1}{L-j-1}\sum_{i=1}^{L-j-1} C_{i,i+j} \; (j = 1, 2, \ldots, \lambda; \lambda < L),$$

here $C_{i,i+j}$ reflects physicochemical properties of any two dinucleotides with a certain distance *j*, and can be calculated as:

$$C_{i,i+j} = \frac{1}{3}\{[P_e(D_i) - P_e(D_{i+j})]^2 + [P_g(D_i) - P_g(D_{i+j})]^2 + [P_f(D_i) - P_f(D_{i+j})]^2\},$$

here, P_e, P_g and P_f represents three physicochemical properties including enthalpy, entropy and free energy (**Table S1**). In addition, $P(D_i)$ represents the physicochemical values of the *i*-th dinucleotide (D_i), similarly, $P(D_{i+j})$ means physicochemical values of the (*i*+*j*)-th dinucleotide. The detailed process of calculating is depicted in Figure S1.

In this study, the parameters *w* and λ are set as 0.9 and 6, respectively according to the application of PseDNC (Chen, Feng, Ding, Lin, & Chou, 2015; Chen, Xing, & Zou, 2017).

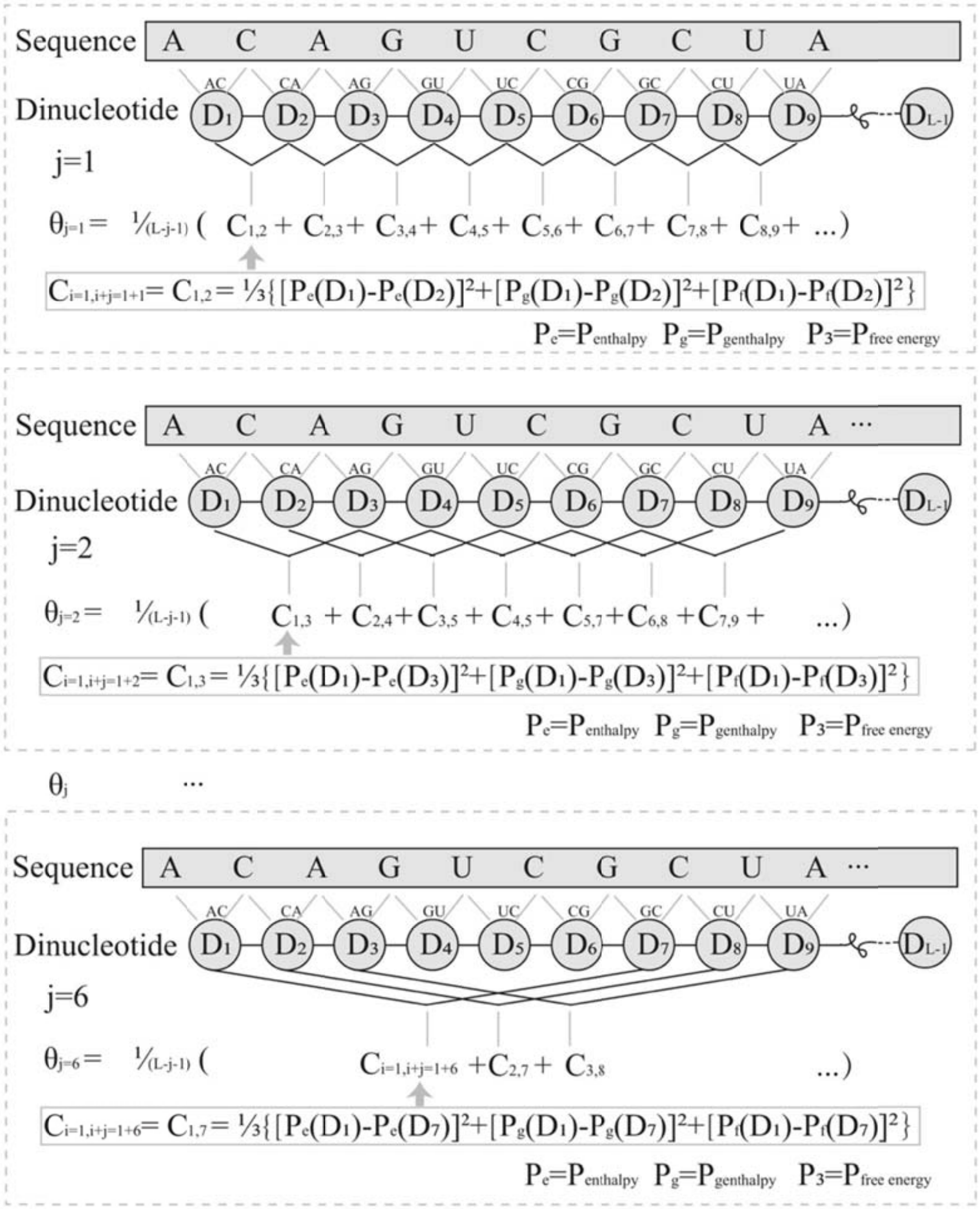

Figure S1. The flowchart of calculating the parameter θ .

Table S1. Physicochemical property values of 16 dinucleotides.

Dinucleotide	Enthalpy (K_a/mol)	Entropy (eU)	Free energy (K_a/mol)
GG	-12.2	-29.7	-3.26
GA	-13.3	-35.5	-2.35
GC	-14.2	-34.9	-3.42
GU	-10.2	-26.2	-2.24
AG	-7.6	-19.2	-2.08
AA	-6.6	-18.4	-0.93
AC	-10.2	-26.2	-2.24
AU	-5.7	-15.5	-1.1
CG	-8	-19.4	-2.36
CA	-10.5	-27.8	-2.11
CC	-12.2	-29.7	-3.26
CU	-7.6	-19.2	-2.08
UG	-7.6	-19.2	-2.11
UA	-8.1	-22.6	-1.33
UC	-10.2	-26.2	-2.35
UU	-6.6	-18.4	-0.93

References:

Chen, W., Feng, P., Ding, H., Lin, H., & Chou, K. C. (2015). iRNA-Methyl: Identifying N(6)-methyladenosine sites using pseudo nucleotide composition. *Anal Biochem, 490*, 26-33. doi:10.1016/j.ab.2015.08.021

Chen, W., Xing, P., & Zou, Q. (2017). Detecting N(6)-methyladenosine sites from RNA transcriptomes using ensemble Support Vector Machines. *Sci Rep, 7*, 40242. doi:10.1038/srep40242
