# Supplementary figures and images for: Transcriptome-Wide Annotation of m5C RNA Modifications Using Machine Learning
Source: Front Plant Sci. 2018 Apr 18;9:519. doi: 10.3389/fpls.2018.00519 (PMC5915569; doi:10.3389/fpls.2018.00519)

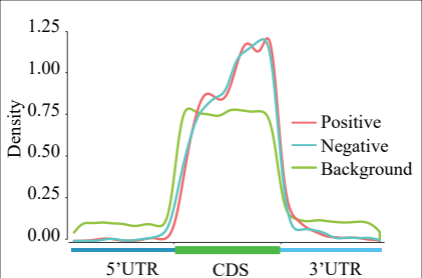

Supplement: Figure S1 — Location distribution of positive, negative and background samples along the 5′-UTR, CDS and 3′-UTR, normalized for transcript length (relative distance). [file Image1.PDF]

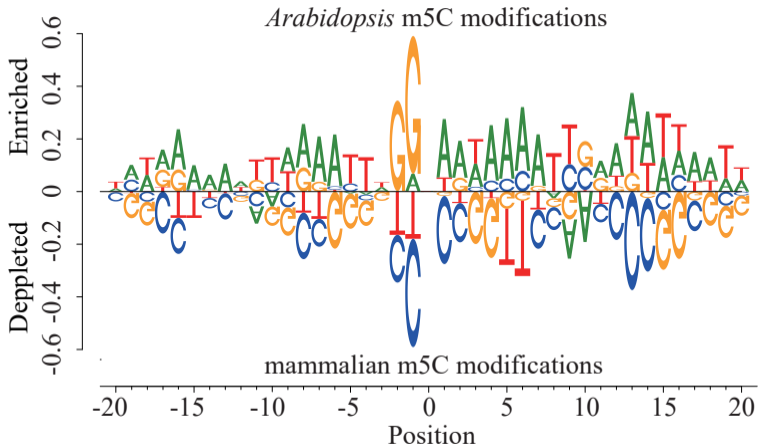

Supplement: Figure S2 — Two sample logos of Arabidopsis and mammalian m5C modifications. It shows nucleotides which are enriched or depleted in the surrounding region of m5C modifications. [file Image2.PDF]

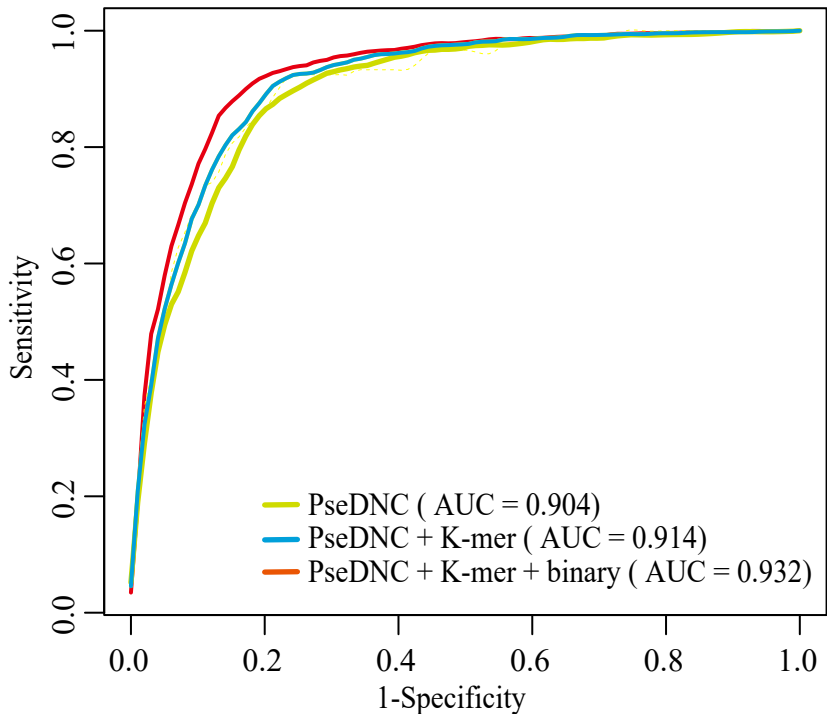

Supplement: Figure S3 — The ROC curve of 10-fold cross-validation illustrating the performance of PEA-m5C with different feature encoding schemes. [file Image3.PDF]

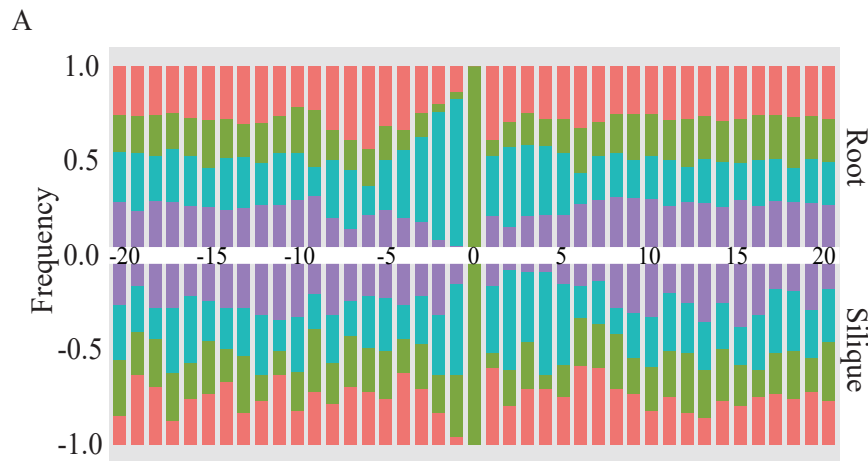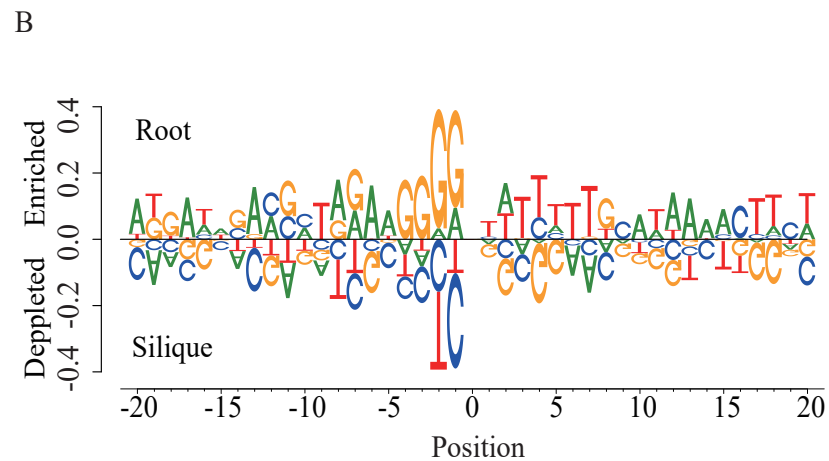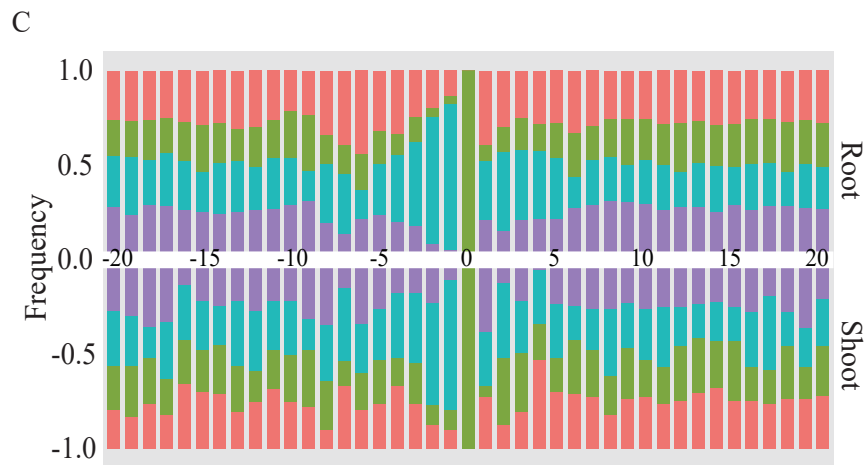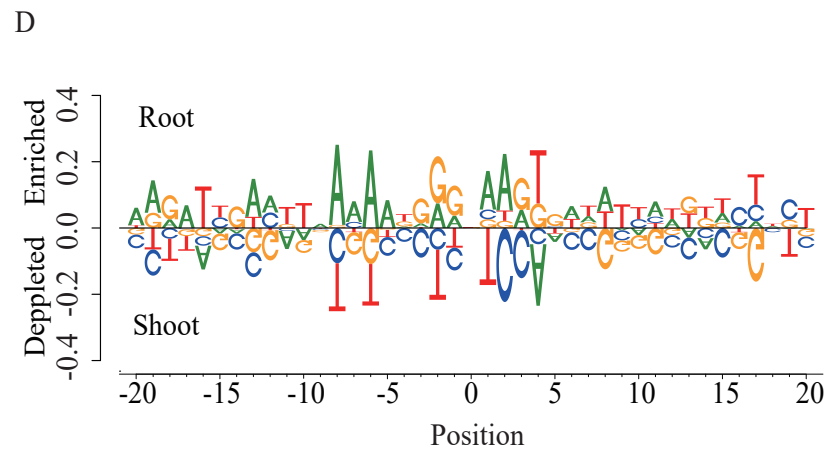

Supplement: Figure S4 — Different sequence patterns of m5C modifications in DatasetHT, DatasetIT1, and DatasetIT2. (A) Frequencies of 41 * 4 position-specific bases in DatasetHT (Root tissue) and DatasetIT1 (Silique tissue). (B) Two sample logos DatasetHT (Root tissue) and DatasetIT1 (Silique tissue) m5C modifications. (C) Frequencies of 41 * 4 position-specific bases in DatasetHT (Root tissue) and DatasetIT2 (Shoot tissue). (D) Two sample logos DatasetHT (Root tissue) and DatasetIT2 (Shoot tissue) m5C modifications. [file Image4.PDF]
